# Food Sources of Dietary Potassium in the Adult Japanese Population: The International Study of Macro-/Micronutrients and Blood Pressure (INTERMAP)

**DOI:** 10.3390/nu12030787

**Published:** 2020-03-17

**Authors:** Nagako Okuda, Akira Okayama, Katsuyuki Miura, Katsushi Yoshita, Naoko Miyagawa, Shigeyuki Saitoh, Hideaki Nakagawa, Kiyomi Sakata, Queenie Chan, Paul Elliott, Hirotsugu Ueshima, Jeremiah Stamler

**Affiliations:** 1 Department of Health and Nutrition, University of Human Arts and Sciences, Saitama 339-8539, Japan; 2 Department of Non-Communicable Diseases, Research Institute of Strategy for Prevention, Tokyo 104-0033, Japan; aokayama@jrisp.com; 3 Department of Public Health, Shiga University of Medical Science, Otsu 520-2192, Japan; miura@belle.shiga-med.ac.jp (K.M.); hueshima@belle.shiga-med.ac.jp (H.U.); 4 Center for Epidemiologic Research in Asia, Shiga University of Medical Science, Otsu 520-2192, Japan; 5 Department of Food Science and Nutrition, Graduate School of Human Life Science and Nutrition, Osaka City University, Osaka 558-8585, Japan; yoshita@life.osaka-cu.ac.jp; 6 International Center for Nutrition and Information, National Institute of Biomedical Innovation, Health and Nutrition, Tokyo 162-8636, Japan; naocom@belle.shiga-med.ac.jp; 7 School of Health Sciences, Sapporo Medical University, Sapporo 060-8556, Japan; ssaitoh@sapmed.ac.jp; 8 Medical Research Institute, Kanazawa Medical University, Kanazawa 920-0265, Japan; hnakagaw@kanazawa-med.ac.jp; 9 Department of Hygiene and Public Health, Iwate Medical University, Yahaba 028-3694, Japan; ksakata@iwate-med.ac.jp; 10 MRC-PHE Centre for Environment and Health, Department of Epidemiology and Biostatistics, School of Public Health, Imperial College London, London SW7 2AZ, UK; q.chan@imperial.ac.uk (Q.C.); p.elliott@imperial.ac.uk (P.E.); 11 Feinberg School of Medicine, Northwestern University, Chicago, IL 60611, USA; j-stamler@northwestern.edu

**Keywords:** nutrition, potassium intake, 24-hr urine, 24-hr dietary recall, Japan, epidemiology

## Abstract

A lower-than-recommended potassium intake is a well-established risk factor for increased blood pressure. Although the Japanese diet is associated with higher sodium intake and lower potassium intake, few studies have examined the source foods quantitatively. Studies on dietary patterns in association with potassium intake will be useful to provide dietary advice to increase potassium intake. Twenty-four-hour (hr) dietary recall data and 24-hr urinary potassium excretion data from Japanese participants (574 men and 571 women) in the International Study of Macro/Micronutrients and Blood Pressure (INTERMAP) were used to calculate food sources of potassium and compare food consumption patterns among quartiles of participants categorized according to 24-hr urinary potassium excretion per unit of body weight (UK/BW). The average potassium intake was 2791 mg/day per participant, and the major sources were vegetables and fruits (1262 mg/day), fish (333 mg/day), coffee and tea (206 mg/day), and milk and dairy products (200 mg/day). Participants in the higher UK/BW quartile consumed significantly more vegetables and fruits, fish, and milk and dairy products, and ate less rice and noodles. Conclusion: Advice to increase the intake of vegetables and fruits, fish, and milk may be useful to increase potassium intake in Japan.

## 1. Introduction

Potassium is the most abundant cation in the human body, with 98% being intracellular and 2% being extracellular. Maintaining normal potassium homeostasis and the appropriate balance of potassium across the cell membrane is essential for cell function: particularly in excitable cells such as muscles and nerves [1]. Epidemiological and clinical studies revealed that a high-potassium diet reduced the blood puressure (BP) of individuals with hypertension and the average BP [2,3,4]. An association between a higher potassium intake and reduced cardiovascular disease (CVD) mortality was demonstrated in prospective cohort studies [3,4]. Based on these findings, the World Health Organization (WHO) recommended that healthy adults consume at least 3510 mg/day of potassium in order to prevent increased BP and CVDs [5], while setting the salt (sodium chloride) reduction target for adults at less than 5 g/day [6]. In Japan, where more than 70% of the elderly population is hypertensive [7], the average daily salt intake level for adults was 9.9 g and that for potassium was 2279 mg according to the National Health and Nutrition Survey Japan 2016 [7], demonstrating an unfavorable high-sodium and low-potassium pattern. 

Vegetables and fruits have been thought to be major sources of potassium, and are often recommended in clinical and health education settings to prevent hypertension [8]. They are also recommended for those with impaired glucose tolerance or dyslipidemia because they are good sources of dietary fiber [9,10]. However, the effects of such education to encourage vegetable and fruit consumption are unclear. The National Health Nutrition Survey reported the declining trend in consumption of vegetables (279 g/day in 2001 to 265 g/day in 2016) and fruit (132 g/day to 99 g/day) [7]. Attention should be paid to other source foods of potassium because it exists in abundance in a wide variety of both animal products and vegetables, and studies on the actual food sources and dietary patterns of those with a lower/higher potassium intake in Japan are scarce. Studies on a high-potassium diet will be useful to create an effective dietary modification strategy to increase potassium intake.

The International Study of Macro/Micronutrients and Blood Pressure (INTERMAP) is an international cooperative study in Japan, the People’s Republic of China, the United Kingdom, and the United States, in which detailed data on dietary intake were obtained from each participant through four 24-hr dietary recalls [11,12]. Two timed 24-hr urine collections were performed to assess urinary sodium and potassium excretion. Using the Japanese results collected between 1996 and 1998 (574 men and 571 women), we examined food intake patterns associated with 24-hr urinary sodium excretion, and found that people with higher sodium excretion ate more Japanese-style foods, including miso-soup, salted fish, and rice, and ate fewer Western foods, i.e., bread and milk [13]. In the present study, we investigate potassium source foods and evaluate differences in food consumption patterns among participants with a higher/lower potassium intake evaluated by 24-hr urinary potassium excretion per unit of body weight (UK/BW).

## 2. Methods

Detailed methods of the INTERMAP Study have been previously described [11,12]. Briefly, participants visited clinics four times on two consecutive days, approximately two to three weeks apart. Anthropometrics, BP measurements, and medical and lifestyle data were collected, including four in-depth 24-hr dietary recalls and two timed 24-hr urine specimens. 

### 2.1. Participants

Participants were men and women aged 40 to 59 years recruited by four research centers in Japan. The four population samples were factory workers from Toyama in central Japan (149 men and 150 women), factory workers from Sapporo in northern Japan (149 men and 148 women), residents of Aito, a rural town in Shiga prefecture, central Japan (130 men and 129 men), and factory workers from Wakayama, central Japan (146 men and 144 women). The ethical committees of the Shiga University of Medical Science, Sapporo Medical University, Kanazawa Medical University, and Wakayama Medical University approved the study protocol. Written informed consent was received from all participants.

### 2.2. Data Collection

BP was measured eight times for each participant (twice at each of the four clinic visits) using a random zero sphygmomanometer by trained observers. Height and weight with light clothes were measured at each visit. Using a questionnaire, trained observers asked about lifestyles and the use of medications.

### 2.3. Dietary Assessment

Four in-depth 24-hr dietary recalls were conducted for each participant, one at each of the four visits, by specially trained dietary interviewers [12]. Prior to the data collection, in cooperation with the INTERMAP international nutritionist, a Japanese nutritionist supervising Japanese dietary data collection trained all interviewers and certified that they had sufficient skills to conduct dietary interviews and process dietary data. Standardized quality control procedures were utilized to ensure the quality of dietary data throughout data collection. Standardized sets of food models were used at all four research centers. In the 24-hr dietary recalls, participants were asked to report all the foods and beverages except for water consumed during the previous 24 hr. The INTERMAP Food Table Japan, an integrated food composition table based on the Standard Tables for Food Composition in Japan, 4th edition, was used to code foods and calculate nutrient intake [14]. Further details about the dietary surveys were described in previous reports. Averages of food and nutrient intakes obtained from four 24-hr recalls per participant were used in the analysis.

### 2.4. Urine Collection, and Urinary Na and K Measurement

Each participant provided two 24-hr urine collections, with start and end times recorded at the research center (at visits 1–2 and 3–4); measurements included urinary volume, Na, K, and creatinine. Eight percent of the urine samples were split locally and sent to the Central Laboratory for blinded estimation of technical error. Urinary values per 24-hr were calculated as the product of urinary concentration and timed volume, standardized to 24 hr. Averages obtained from two 24-hr urine samples per participant were used in the analysis.

### 2.5. Data Analysis

To explore the association of food consumption with 24-hr urinary potassium excretion in the Japanese participants, 2508 food codes used in the dietary recalls were classified into 29 groups for this study (Table 1). For example, potassium is more abundant in muscle than in adipose tissue in animals; therefore, meat was categorized as “with visible fat” or “without visible fat”. Fish was grouped according to total fat content; <8% or >=8%. The average content of potassium and sodium (mg/100 g) for each food group was calculated as the total potassium amount divided by the total food amount consumed in all dietary recalls. Food (g) and potassium (mg) intakes per day and per 1000 kcal of the 29 food groups were calculated for each participant. Calculations were also made for sodium. Subtotals for food categories (i.e., starchy foods, fish) were calculated.

The mean of the two 24-hr urinary potassium excretion values divided by body weight (UK/BW, mmoL/24 hr/kg) was calculated for each participant. Participants were divided into quartiles according to UK/BW, men, and women separately. Potassium intake from each of the 29 food groups (mg/1000 kcal) was compared among the quartiles of UK/BW, men, and women combined. We used the potassium excretion value adjusted by body weight in order to examine the characteristics of the food intake pattern by excluding the effects of the amount eaten or body size.

All eight readings of systolic and diastolic BP (SBP and DBP) were used to calculate the average SBP/DBP of each participant. For height and weight, means of four measurements were used in the analyses. Body mass index (BMI) was calculated as weight (kg) divided by the height squared (m). The *p*-value was calculated to examine the linear relationship among the UK/BW quartiles, analyzed as a continuous variable. For categorical variables, the chi-squared test or Fisher’s exact test was used. Two-tailed significance level was set at *p* < 0.05. SPSS statistics version 21.0 (IBM Corporation, Tokyo, Japan) was used for all analyses.

## 3. Results

The food groups, their average potassium and sodium content, and the average daily intake from these foods are shown in Table 1. The average potassium content was high in both animal products (335 mg/100 g for fish, and 291 mg/100 g for meat), and vegetables and fruits (262 mg/100 g). Starchy foods had a low average potassium content (35 mg/100 g). The average total dietary potassium intake per participant estimated from the dietary survey was 2791 mg/day. Major potassium source foods were vegetables and fruits (1262 mg/day of potassium), milk, beverages, sweets and snacks (561 mg/day), and fish (333 mg/day). The potassium content in salted foods (salted fish, cured meat, and salted vegetables) was similar to that in non-salted foods, but the sodium content was high and its intake from these foods was 1315 mg/day and accounted for 28% of the total sodium intake.

Characteristics of participants according to UK/BW quartiles are shown in Table 2. For both men and women, the mean age was significantly higher and average BMI was significantly lower in the higher quartile. There was no significant difference in drinking habits among quartiles. Fewer current smokers were found in the higher UK/BW quartile among men, but not among women. Few participants reported following vegetarian or reduced salt diets, and there were no significant differences among quartiles. We found no significant differences in the use of medications for hypertension, diabetes mellitus, or lipid disorders among UK/BW quartiles. The average SBP and DBP were significantly lower in the higher quartile in both men and women.

Urinary excretion of potassium and sodium according to quartiles of UK/BW are shown in Table 3. In total, 24-hr urinary potassium excretion level was higher in women than in men, but sodium excretion and urinary Na/K ratio was higher in men. Although 24-hr urinary sodium excretion was moderately higher in the higher quartile, the urinary sodium to potassium ratio was significantly lower in the higher quartile due to significantly higher potassium excretion. 

The potassium intake per 1000 kcal of total energy intake from food groups with participants stratified by UK/BW quartiles is shown in Table 4. The potassium intake from vegetables and fruits, fish, milk, and miso soup was significantly higher in the higher quartiles. Coffee and tea were a source of potassium (102 mg/1000 kcal) equivalent to fruits (107 mg/1000 kcal) or milk (102 mg/1000 kcal), but there was no significant difference in potassium contribution among quartiles. 

## 4. Discussion

We examined dietary patterns associated with 24-hr urinary potassium excretion using data from the detailed dietary survey in the INTERMAP Japan Study, and quantitatively demonstrated the relationship between food intake and potassium excretion. To the best of our knowledge, this is the first study in which participants were categorized according to 24-hr urinary potassium excretion and potassium intake from detailed food groups was compared. Major potassium source foods were vegetables and fruits, fish, and milk. Consumption of these foods was higher in the participants with higher potassium excretion. Potassium from vegetables and fruits accounted for almost half of the daily potassium intake.

One study in Spain found a higher vegetable and fruit intake in the higher 24-hr urinary potassium excretion group, but the potassium intake from these foods was not presented [15]. Previously, we used results from the National Health and Nutrition Survey Japan in 1980 and 1990, and compared food intakes among those with a higher/lower potassium intake estimated from household-based semi-weighed dietary records [16]. The food intake pattern associated with a higher potassium intake was similar to that in the present study, i.e., lower intake from cereals, and higher intake from vegetables, fruits, fish, and milk and dairy products. A recent study in Poland used Household Budget Survey data, and reported vegetables, meat and meat products, and cereal products to be the major potassium source foods [17]. 

In this study, participants with a lower UK/BW consumed more rice and noodles and had a higher 24-hr urinary Na/K ratio. Dietary habits of having more simple meals, such as noodles or rice bowl dishes, may lead to low potassium intake. Conversely, those with a higher UK/BW consumed more vegetables, fruits, and fish. Fish and vegetables often require a longer cooking process than meat. People with a higher potassium intake may have eaten vegetables and fish habitually by taking time and effort in food preparation. For those with lower potassium intake, an economical and effortless eating style, e.g., pre-cut fruit or vegetables, may be useful to improve their consumption.

Some other foods to increase the potassium intake should be recommended to prevent hypertension and CVDs. Studies have reported a lower prevalence of hypertension and CVD mortality among people with higher milk consumption [18,19]. Although the prevalence of lactose intolerance is reported to be as high as 90% in Asian populations [20], there are few symptomatic individuals with moderate milk consumption. The percentage of milk wasted from elementary school lunch was 8.5% [21], and 28% of adults reported having abdominal symptoms after drinking milk [22]. Milk may be a good source of potassium for those without abdominal symptoms because it is generally low-energy and requires no additional sodium.

Another option may be the use of condiments with potassium-enriched salt, which may reduce sodium intake and improve the dietary Na/K ratio [23,24,25]. The major source of sodium in Japan is soy sauce, which we previously reported to account for 806 mg of the daily sodium intake [13]. Habitual use of potassium-enriched soy sauce, miso, and other prepared foods will be useful for sodium reduction and potassium increase, without advising people to improve their diet. Participants in an intervention study using potassium-enriched condiments in Japan successfully used these condiments at home while cooking and their urinary Na/K ratio decreased significantly [25]. Coffee and tea are infusions of coffee beans or tea leaves, respectively, and provide an amount of potassium (205 mg/day) nearly equal to that of one serving of vegetable dish. Potassium intake from fruit juice, other beverages (carbonated drinks and sports drinks), alcoholic beverages, and sweets and snacks was small. These foods were not good sources of potassium. Excess consumption of these foods, including ultra-processed foods, may lead to obesity and related outcomes through a higher energy intake [26]. 

The average SBP and DBP were significantly lower in the higher UK/BW quartile. Although previous studies have reported lower BP levels in people with a higher potassium intake [2,4], our results may be related to the lower average BMI in the higher UK/BW quartile. They consumed more vegetables, fruits, and fish, and ate less starchy foods and meat. These dietary patterns with a higher intake of low-energy-density foods may have led to the overall lower BMI level.

Our study had several strengths. Highly standardized procedures were adopted for 24-hr dietary recall surveys in the INTERMAP Study, and we obtained highly detailed food and beverage consumption data of individual participants. Thus, we were able to evaluate potassium source foods quantitatively, including coffee and tea, which are often excluded in dietary surveys. In addition, 24-hr urine was collected during the period of the 24-hr recall survey; a large portion of the potassium intake during the 2nd and 4th 24-hr dietary recalls should have been excreted into the 1st and 2nd 24-hr urine samples, respectively. The correlation coefficient between dietary and urinary estimates of potassium in this survey was 0.565 [9], and the dietary data were assumed to be sufficient to examine the food intake factors associated with the difference in urinary potassium excretion. One limitation of our study is that the data were collected from 1996 to 1998, and dietary habits may have changed, specifically the increase in eating-out and use of prepared foods. To examine the current status of potassium intake, further studies using an extended food database that covers foods served in restaurants and prepared foods should be performed.

In summary, people with a higher dietary potassium intake consumed more vegetables, fruits, fish, and milk, and ate less rice and noodles in Japan. Advice to increase the intake of vegetables, fruits, fish, and milk may be useful to increase potassium intake.

## Figures and Tables

**Table 1:** Twenty-nine food categories for investigation of the association of food group consumption with potassium (K) intake.

Food Group	Food Intake (g/Day/Participant)	Average Content (mg/100g)		Average Intake (mg/Day/Participant)	
		K	Na	K	Na
	Mean ± SD			Mean ± SD	Mean ± SD
**Starchy foods, total**	522 ± 181	35	57	182 ± 60	297 ± 265
Rice	410 ± 181	28	2	113 ± 50	9 ± 10
Bread	33 ± 36	100	523	33 ± 37	171 ± 191
Noodles without soup	72 ± 75	37	146	26 ± 33	106 ± 189
Other cereals	7 ± 8	132	163	10 ± 12	12 ± 19
**Fish and fish products, total**	99 ± 56	335	799	333 ± 201	795 ± 554
Fish, fat <8% (not salted)	42 ± 39	373	248	155 ± 142	103 ± 121
Fish, fat >=8% (not salted)	18 ± 19	366	252	65 ± 73	45 ± 89
Salted fish	40 ± 29	282	1613	113 ± 100	647 ± 520
**Meat, meat products and eggs, total**	58 ± 40	291	392	168 ± 111	213 ± 179
Meat, without skin/visible fat	20 ± 27	311	57	63 ± 73	12 ± 18
Meat, with skin/visible fat	26 ± 23	307	59	78 ± 72	15 ± 15
Cured meat product	12 ± 16	222	1089	27 ± 34	130 ± 164
Other manufactured foods (e.g., precooked hamburgers)	15 ± 24	219	619	34 ± 49	95 ± 157
Eggs	39 ± 25	118	147	46 ± 29	57 ± 45
**Vegetable and fruits, total**	481 ± 194	262	131	1262 ± 520	628 ± 489
Vegetables	230 ± 97	304	30	699 ± 327	70 ± 98
Salted vegetables (Japanese pickles)	29 ± 26	310	1864	90 ± 92	538 ± 471
Potatoes	49 ± 39	377	5	186 ± 168	2 ± 2
Soy beans, legumes and nuts	60 ± 51	133	18	80 ± 77	11 ± 22
Fruits, fresh, dried and canned	113 ± 102	159	6	206 ± 190	7 ± 31
**Milk, beverages, and sweets and snacks, total**	1138 ± 470	49	18	561 ± 251	200 ± 125
Milk and dairy products	126 ± 117	158	76	200 ± 190	96 ± 87
Fruit juice	10 ± 30	183	6	9 ± 34	1 ± 3
Coffee, tea	698 ± 348	93	3	205 ± 122	20 ± 28
Alcohol beverages	233 ± 335	30	3	70 ± 114	7 ± 10
Other beverages (carbonated drinks and sports drinks)	30 ± 79	51	43	15 ± 44	13 ± 38
Sweets and snacks	40 ± 35	154	159	61 ± 62	64 ± 69
**Soup and condiments, total**	244 ± 109	84	992	206 ± 93	2426 ± 782
Miso soup	127 ± 89	59	340	74 ± 70	431 ± 301
Soup for noodles	45 ± 53	44	546	20 ± 26	247 ± 305
Salty condiments and spices (e.g., soy sauce, salt, dressings)	49 ± 30	224	3558	109 ± 58	1736 ± 642
Sugar and sweeteners	12 ± 7	15	1	2 ± 8	0 ± 0
Fats and oils	12 ± 7	6	95	1 ± 1	11 ± 24
TOTAL	2597 ± 601			2791 ± 701	4650 ± 1279

**Table 2:** Characteristics of participants stratified by quartiles of 24-hr urinary potassium (K) excretion per unit of body weight (mmoL/24 hr/kg, UK/BW), men and women, ages 40–59.

	Q1	Q2	Q3	Q4	*P*
	Mean ± SD	Mean ± SD	Mean ± SD	Mean ± SD	
**Men**					
n	143	144	144	143	
Age (year)	49.2 ± 5.8	48.8 ± 5	49.7 ± 5.1	50.4 ± 5.2	0.02
Body mass index (kg/m^2^)	24.4 ± 2.9	24.1 ± 2.7	23.6 ± 2.7	22.8 ± 2.3	<0.01
Drinking habits, n (%)					
Current drinker	138 (96.5)	139 (96.5)	140 (97.2)	141 (98.6)	0.59
Ex-drinker	0 (0)	2 (1.4)	1 (0.7)	1 (0.7)	
Non-drinker	5 (3.5)	3 (2.1)	3 (2.1)	1 (0.7)	
Smoking habits, n (%)					
Current smoker	82 (57.3)	79 (54.9)	69 (47.9)	67 (46.9)	0.006
Ex-smoker	44 (30.8)	29 (20.1)	37 (25.7)	34 (23.8)	
Non-smoker	17 (11.9)	36 (25)	38 (26.4)	42 (29.4)	
On vegetarian diet, n (%)	0 (0)	1 (0.7)	2 (1.4)	0 (0)	0.62
On reduced salt diet, n (%)	4 (2.8)	1 (0.7)	4 (2.8)	5 (3.5)	0.27
Use of medication, n (%)					
Hypertension	13 (9.1)	10 (6.9)	6 (4.2)	5 (3.5)	0.16
Diabetes mellitus	1 (0.7)	3 (2.1)	2 (1.4)	4 (2.8)	0.16
Lipid disorder	4 (2.8)	3 (2.1)	3 (2.1)	8 (5.6)	0.12
SBP (mmHg)	123.2 ± 13.1	120.2 ± 12.9	119.5 ± 12.6	118.7 ± 12.7	0.003
DBP (mmHg)	78.8 ± 10.4	77.2 ± 9.9	76.3 ± 8.7	74.9 ± 10.6	0.001
**Women**					
n	142	143	143	143	
Age (year)	48.3 ± 5.4	48.9 ± 5.2	48.9 ± 5.2	50.7 ± 5.2	<0.001
Body mass index (kg/m^2^)	24.4 ± 3.4	23.5 ± 3	22.7 ± 3	22.1 ± 2.3	<0.001
Drinking habits, n (%)					
Current drinker	117 (82.4)	118 (82.5)	126 (88.1)	120 (83.9)	0.38
Ex-drinker	4 (2.8)	5 (3.5)	4 (2.8)	9 (6.3)	
Non-drinker	21 (14.8)	20 (14)	13 (9.1)	14 (9.8)	
Smoking habits, n (%)					
Current smoker	18 (12.7)	14 (9.8)	8 (5.6)	9 (6.3)	0.31
Ex-smoker	3 (2.1)	3 (2.1)	5 (3.5)	2 (1.4)	
Non-smoker	121 (85.2)	126 (88.1)	130 (90.9)	132 (92.3)	
On vegetarian diet, n (%)	1 (0.7)	2 (1.4)	1 (0.7)	2 (1.4)	1.00
On reduced salt diet, n (%)	2 (1.4)	9 (6.3)	6 (4.2)	5 (3.5)	0.32
Use of medication, n (%)					
Hypertension	13 (9.2)	8 (5.6)	9 (6.3)	7 (4.9)	0.11
Diabetes mellitus	2 (1.4)	2 (1.4)	4 (2.8)	0 (0)	0.32
Lipid disorder	6 (4.2)	4 (2.8)	4 (2.8)	5 (3.5)	0.87
SBP (mmHg)	115.5 ± 13.9	115.2 ± 14.9	113.4 ± 13.2	112.2 ± 13.5	0.026
DBP (mmHg)	71.5 ± 10.4	71.2 ± 9.7	69.8 ± 9.2	69.2 ± 9	0.021

*P*-values, obtained from trend analysis or chi-squared tests. SBP, systolic blood pressure; DBP, diastolic blood pressure.

**Table 3:** Electrolyte excretion from 24-hr urine of participants stratified by quartiles of 24-hr urinary potassium excretion per unit of body weight (mmoL/24 hr/kg, UK/BW), men and women, ages 40–59.

	Q1	Q2	Q3	Q4	Trend *P*
	Mean ± SD	Mean ± SD	Mean ± SD	Mean ± SD	
**Men**					
K per body weight (mmoL/24 hr/kg)	0.52 ± 0.07	0.67 ± 0.03	0.78 ± 0.03	1.00 ± 0.17	<0.001
K (mmoL/24 hr)	36.1 ± 6.6	45.3 ± 6.6	52 ± 7.1	63.6 ± 13.2	<0.001
Na per body weight (mmoL/24 hr/kg)	2.74 ± 0.71	3.09 ± 0.71	3.3 ± 0.67	3.54 ± 0.93	<0.001
Na (mmoL/24 hr)	188.3 ± 51.4	208.7 ± 52.3	220.6 ± 52.1	224.5 ± 63.3	<0.001
Na/K (mmoL/mmol)	5.33 ± 1.43	4.67 ± 1.11	4.3 ± 0.9	3.61 ± 0.94	<0.001
**Women**					
K per body weight (mmoL/24 hr/kg)	0.59 ± 0.08	0.77 ± 0.05	0.94 ± 0.05	1.24 ± 0.19	<0.001
K (mmoL/24 hr)	34.5 ± 6.3	43.2 ± 6.5	50.9 ± 7.4	65.3 ± 11.2	<0.001
Na per body weight (mmoL/24 hr/kg)	2.82 ± 0.79	3.36 ± 0.74	3.56 ± 0.86	3.76 ± 0.98	<0.001
Na (mmoL/24 hr)	166.5 ± 54.7	187.8 ± 49.4	192.5 ± 51.6	197.1 ± 51.9	<0.001
Na/K (mmoL/mmoL)	4.91 ± 1.3	4.4 ± 1	3.84 ± 0.9	3.11 ± 0.87	<0.001

**Table 4:** Potassium intake (mg/1000 kcal) from 29 food groups, participants stratified by quartiles of 24-hr urinary potassium excretion per unit of body weight (mmoL/24 hr/kg, UK/BW), men and women, ages 40–59.

	Q1	Q2	Q3	Q4	Trend *P*	Total
	Mean ± SD	Mean ± SD	Mean ± SD	Mean ± SD		Mean ± SD
**Starchy foods, total**	95 ± 25	94 ± 24	86 ± 21	84 ± 23	<0.001	90 ± 24
Rice	58 ± 20	57 ± 20	54 ± 19	53 ± 21	<0.001	55 ± 20
Bread	17 ± 19	17 ± 19	16 ± 18	16 ± 19	0.37	17 ± 19
Noodles	15 ± 18	15 ± 16	12 ± 16	10 ± 14	<0.001	13 ± 16
Other cereals	5 ± 6	5 ± 5	4 ± 4	5 ± 8	0.12	5 ± 6
**Fish and fish products, total**	148 ± 77	161 ± 97	168 ± 83	177 ± 90	<0.001	164 ± 88
Fish, fat <8%	68 ± 58	75 ± 71	79 ± 60	85 ± 71	0.002	77 ± 66
Fish, fat >=8%	30 ± 34	31 ± 34	35 ± 35	30 ± 33	0.54	31 ± 34
Salted fish	50 ± 39	55 ± 44	55 ± 48	62 ± 51	0.002	55 ± 46
**Meat, meat products and eggs, total**	125 ± 57	123 ± 54	118 ± 48	116 ± 59	0.03	120 ± 55
Meat, without visible fat	31 ± 35	28 ± 34	28 ± 32	36 ± 44	0.10	31 ± 36
Meat, with visible fat	40 ± 33	40 ± 35	39 ± 30	32 ± 28	0.005	38 ± 32
Cured product meat	12 ± 15	14 ± 19	12 ± 15	13 ± 17	0.84	13 ± 16
Other manufactured foods	18 ± 26	17 ± 22	16 ± 22	13 ± 22	0.011	16 ± 23
Eggs	24 ± 14	23 ± 14	22 ± 13	22 ± 13	0.023	23 ± 13
**Vegetable and fruits, total**	511 ± 210	583 ± 233	660 ± 246	793 ± 283	<0.001	637 ± 265
Vegetables	291 ± 134	331 ± 157	362 ± 149	426 ± 189	<0.001	352 ± 166
Salted vegetables	41 ± 40	42 ± 44	47 ± 53	48 ± 44	0.029	44 ± 45
Potatoes	72 ± 64	84 ± 76	98 ± 87	117 ± 90	<0.001	93 ± 82
Soy beans and legumes	35 ± 32	37 ± 31	41 ± 36	48 ± 56	<0.001	40 ± 40
Fruits, fresh, dried and canned	72 ± 81	88 ± 84	113 ± 96	153 ± 119	<0.001	107 ± 101
**Beverages, sweets and snacks, total**	260 ± 113	273 ± 121	279 ± 110	306 ± 133	<0.001	280 ± 121
Milk and dairy products	84 ± 92	94 ± 95	101 ± 92	131 ± 109	<0.001	102 ± 99
Coffee, tea	102 ± 59	109 ± 64	99 ± 49	100 ± 64	0.36	102 ± 59
Fruit juice	6 ± 22	4 ± 13	6 ± 18	5 ± 17	0.88	5 ± 18
Other beverages	9 ± 24	8 ± 24	6 ± 17	7 ± 20	0.23	8 ± 21
Sweets and snacks	30 ± 28	29 ± 27	32 ± 32	31 ± 31	0.44	30 ± 30
Alcohol beverages	31 ± 48	29 ± 46	36 ± 50	32 ± 55	0.43	32 ± 50
**Soup and condiments, total**	95 ± 44	101 ± 41	106 ± 44	107 ± 44	<0.001	102 ± 43
Miso soup	30 ± 31	34 ± 33	40 ± 36	45 ± 38	<0.001	37 ± 35
Soup for noodles	11 ± 13	11 ± 12	10 ± 14	9 ± 13	0.02	10 ± 13
Salty condiments and spices	54 ± 26	54 ± 26	55 ± 28	51 ± 21	0.37	54 ± 25
Sugar and sweeteners	1 ± 2	1 ± 4	0 ± 2	2 ± 7	0.017	1 ± 4
Fats and oils	0 ± 1	0 ± 1	0 ± 1	0 ± 1	0.62	0 ± 1
